# Immature human engineered heart tissues engraft in a guinea pig chronic injury model

**DOI:** 10.1242/dmm.049834

**Published:** 2023-06-05

**Authors:** Constantin von Bibra, Aya Shibamiya, Andrea Bähr, Birgit Geertz, Maria Köhne, Tim Stuedemann, Jutta Starbatty, Verena Horneffer-van der Sluis, Ulrich C. Klostermeier, Nadja Hornaschewitz, Xinghai Li, Eckhard Wolf, Nikolai Klymiuk, Markus Krane, Christian Kupatt, Bernhard Hiebl, Thomas Eschenhagen, Florian Weinberger

**Affiliations:** ^1^ Department of Experimental Pharmacology and Toxicology, University Medical Center Hamburg-Eppendorf, 20246 Hamburg, Germany; ^2^ German Centre for Cardiovascular Research (DZHK), partner site Hamburg/Kiel/Lübeck, Germany; ^3^ Medizinische Klinik und Poliklinik, University Clinic Rechts der Isar, Technical University Munich, Germany; ^4^ German Centre for Cardiovascular Research (DZHK), partner site Munich, 80333 München, Germany; ^5^ Institute of Clinical Chemistry and Laboratory Medicine, University Medical Center Hamburg-Eppendorf, 20246 Hamburg, Germany; ^6^ Department of Cardiovascular Surgery, German Heart Centre Munich, Technical University Munich, 80333 München, Germany; ^7^ Gene Center and Center for Innovative Medical Models (CiMM), LMU Munich, 85764 Oberschleißheim, Germany; ^8^ Division of Cardiac Surgery, Yale School of Medicine, New Haven, CT 06510, USA; ^9^ Institute for Animal Hygiene, Animal Welfare and Farm Animal Behaviour, University of Veterinary Medicine Hannover, 30559 Hannover, Germany

**Keywords:** Cardiac repair, Cell transplantation, Chronic injury model, Engineered heart tissue, Heart failure, Tissue engineering

## Abstract

Engineered heart tissue (EHT) transplantation represents an innovative, regenerative approach for heart failure patients. Late preclinical trials are underway, and a first clinical trial started recently. Preceding studies revealed functional recovery after implantation of *in vitro*-matured EHT in the subacute stage, whereas transplantation in a chronic injury setting was less efficient. When transplanting matured EHTs, we noticed that cardiomyocytes undergo a dedifferentiation step before eventually forming structured grafts. Therefore, we wanted to evaluate whether immature EHT (EHT^Im^) patches can be used for transplantation. Chronic myocardial injury was induced in a guinea pig model. EHT^Im^ (15×10^6^ cells) were transplanted within hours after casting. Cryo-injury led to large transmural scars amounting to 26% of the left ventricle. Grafts remuscularized 9% of the scar area on average. Echocardiographic analysis showed some evidence of improvement of left-ventricular function after EHT^Im^ transplantation. In a small translational proof-of-concept study, human scale EHT^Im^ patches (4.5×10^8^ cells) were epicardially implanted on healthy pig hearts (*n*=2). In summary, we provide evidence that transplantation of EHT^Im^ patches, i.e. without precultivation, is feasible, with similar engraftment results to those obtained using matured EHT.

## Teaser

**Summary:** We provide evidence that transplantation of immature engineered heart tissue (EHT) patches without precultivation is feasible, with similar, or even slightly better, engraftment results than those obtained with matured EHT.

## INTRODUCTION

Myocardial injury is followed by a remodeling cascade aiming to compensate for the irreversible loss of cardiomyocytes (CMs). Current therapeutic approaches are not able to address the loss of myocytes, and heart failure is still a major problem after myocardial injury (Khan et al., 2020). New therapeutic approaches are therefore required (Weinberger and Eschenhagen, 2021). Regenerative therapies have been applied successfully in preclinical models (Weinberger et al., 2016; Querdel et al., 2021; Shiba et al., 2012, 2016; Caspi et al., 2007), and first clinical trials with human induced pluripotent stem cell (hiPSC)-derived CMs were initiated, e.g. BioVAT-HF. Transplantation of engineered cardiac constructs, e.g. engineered heart tissue (EHT), has become a meaningful therapeutic attempt. For this strategy, hiPSC-derived CMs are embedded in a three-dimensional matrix and cultured over several weeks before transplantation. During the culture period, CMs mature considerably, coherently start to beat and, at the time of transplantation, resemble human myocardium, both morphologically (e.g. cell alignment and sarcomeric organization) and physiologically (e.g. action potential characteristics, response to pharmacological stimuli) (Querdel et al., 2021; Shadrin et al., 2017; Tiburcy et al., 2017). EHTs engrafted in the injured heart in a dose-dependent manner and improved left-ventricular function in the highest dose (Querdel et al., 2021). The data indicate that a substantial part of the scar must be remuscularized for a meaningful effect. Yet, the beneficial effect was only seen in a subacute setting. EHT transplantation in the clinically more relevant chronic injury model was less efficient (Shiba et al., 2014; Fernandes et al., 2010), both in terms of graft formation and functional recovery (von Bibra et al., 2022). While analyzing graft structure at early time points after transplantation, we noticed a temporary deterioration in CM structure. Over time, CM structure recovered, and the cells formed human grafts with advanced cell organization. We therefore hypothesized that it is feasible to circumvent the cultivation process and transplant non-contracting immature EHT (EHT^Im^) patches.

## RESULTS

### Generation of EHT patches

We examined whether EHT^Im^ patches could engraft in a chronic injury setting. In accordance with previous studies, EHT patches were cast with 15×10^6^ cells (Querdel et al., 2021; von Bibra et al., 2022) [troponin t (TNNT) positivity, 94±2%; *n*=7] and used for transplantation directly (2-5 h) after casting. CMs within the EHT^Im^ patches were immature (e.g. indicated by their small size, round shape) and showed no structured sarcomere expression (Fig. S1A). Human-scale EHT patches for the pig experiments were cast with 4.1-4.8×10^8^ cells (troponin t positivity, 91±0%; *n*=2) and transplanted within 12 h after casting. To compare the cell number at the time of transplantation with previous studies in which matured EHTs were transplanted after a culture period of 3 weeks, we applied two methods: (1) cell number per EHT was assessed by histology, and (2) total DNA content per EHT was measured. Both strategies showed a significant drop in cell number (∼50%), indicating that substantially more cells were transplanted in the current study than in previous studies with matured/beating EHT patches (Fig. S1A-D).

### Engraftment of EHT^Im^

**Fig. 1.**
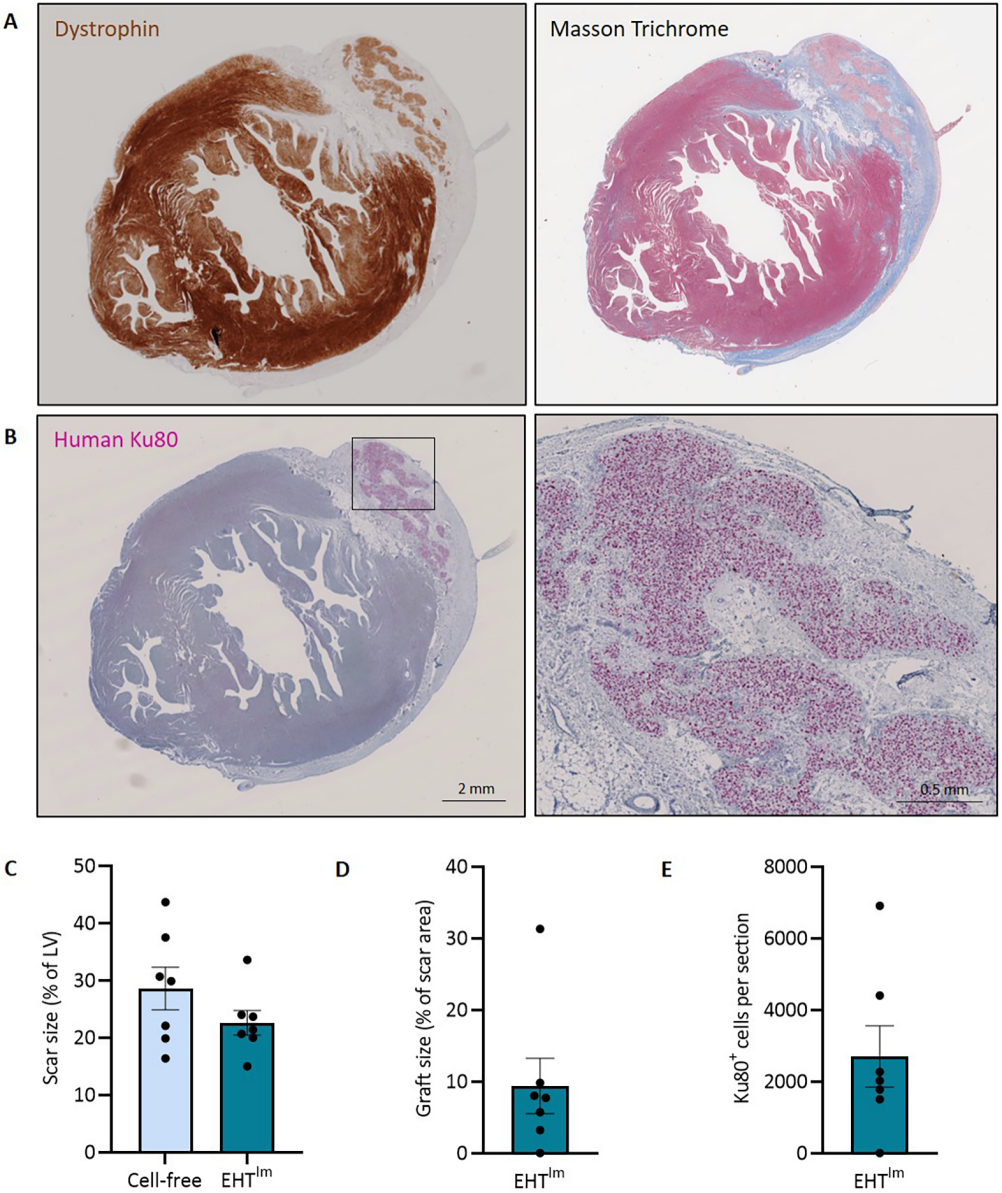
**Histological assessment of guinea pig hearts after EHT^Im^ transplantation.** (A,B) Sections of one heart 4 weeks after transplantation stained for dystrophin and Masson trichrome (A), and human Ku80 (graft shown at higher magnification on the right) (B). (C,D) Quantification of scar size (C) and graft size (D). (E) Quantification of Ku80^+^ cells per section. Each data point represents one heart. Mean±s.e.m. values are shown. EHT^Im^, immature engineered heart tissue; LV, left ventricle.

Four weeks after the induction of myocardial damage, a rethoracotomy was performed, and the scar was covered with an EHT^Im^ or cell-free patch (*n*=7, respectively). After a follow up period of 4 weeks, hearts were excised and prepared for histological analysis (Fig. S2A). Histomorphometry exhibited similar scar areas in the cell-free control group (29±4% of the left ventricle, *n*=7) and the intervention group (23±2%; *n*=7; Fig. 1A,C). Six of seven hearts in the EHT^Im^ group contained engrafted CMs. Human origin of the grafts was demonstrated by human Ku80 (also known as XRCC5) immunostaining (Fig. 1B). The graft area amounted to 9±4% of the total scar area (Fig. 1D), correlating to 2700±860 human cells per section (Fig. 1E).

**Fig. 2.**
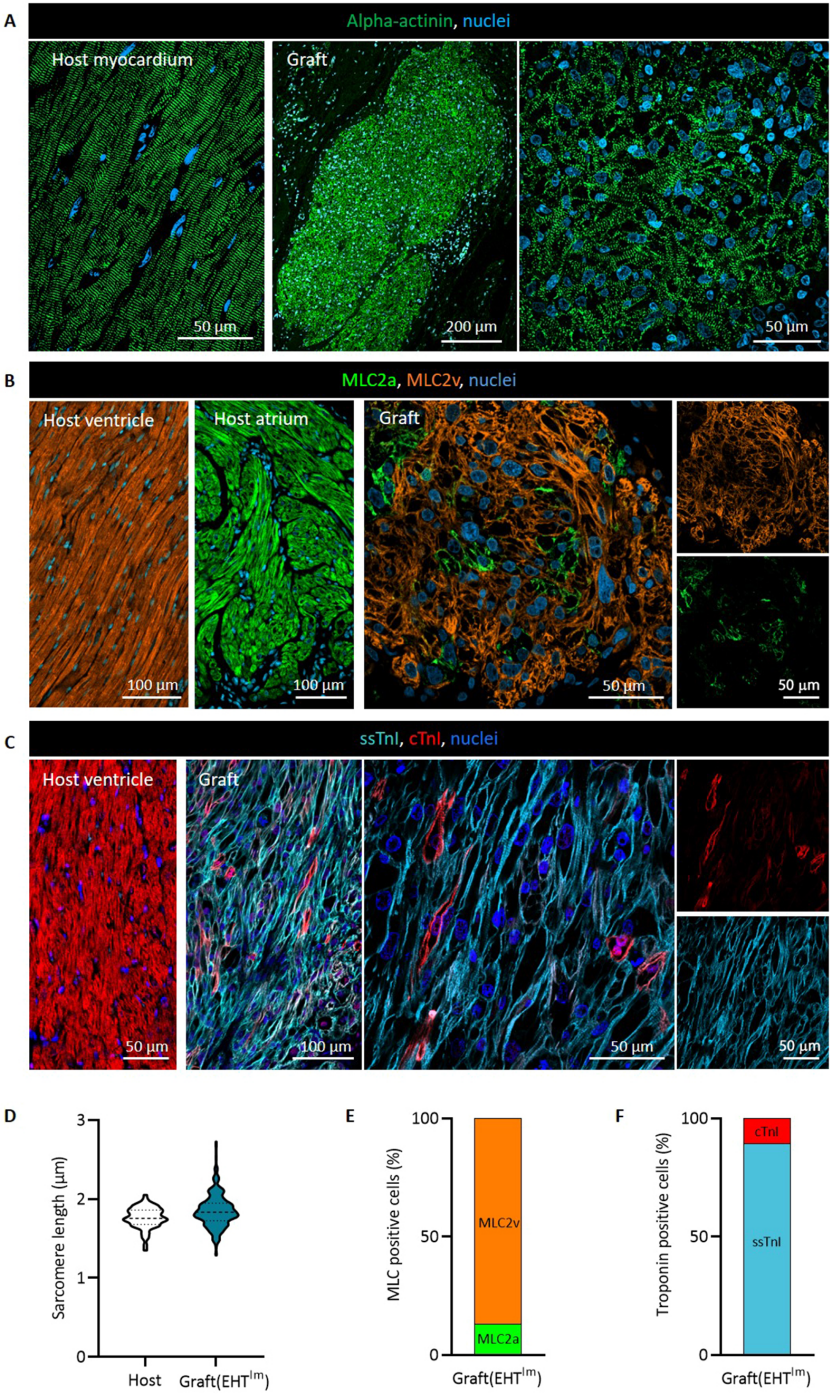
**Structural analysis of engrafted cardiomyocytes.** (A-C) Alpha-actinin (A), (B) myosin light chain (MLC2; B) isoform and (C) troponin I (TnI; C) isoform staining of host and graft myocardium. (D) Quantification of sarcomere length in human grafts compared to guinea pig host myocardium in alpha-actinin-stained sections (*n*=3 hearts/>90 sarcomeres per graft or host myocardium). (E) Quantification of atrial (MLC2a) and ventricular (MLC2v) isoform expression. (F) Quantification of slow skeletal (ssTnI) and cardiac troponin I (cTnI) isoform expression.

**Fig. 3.**
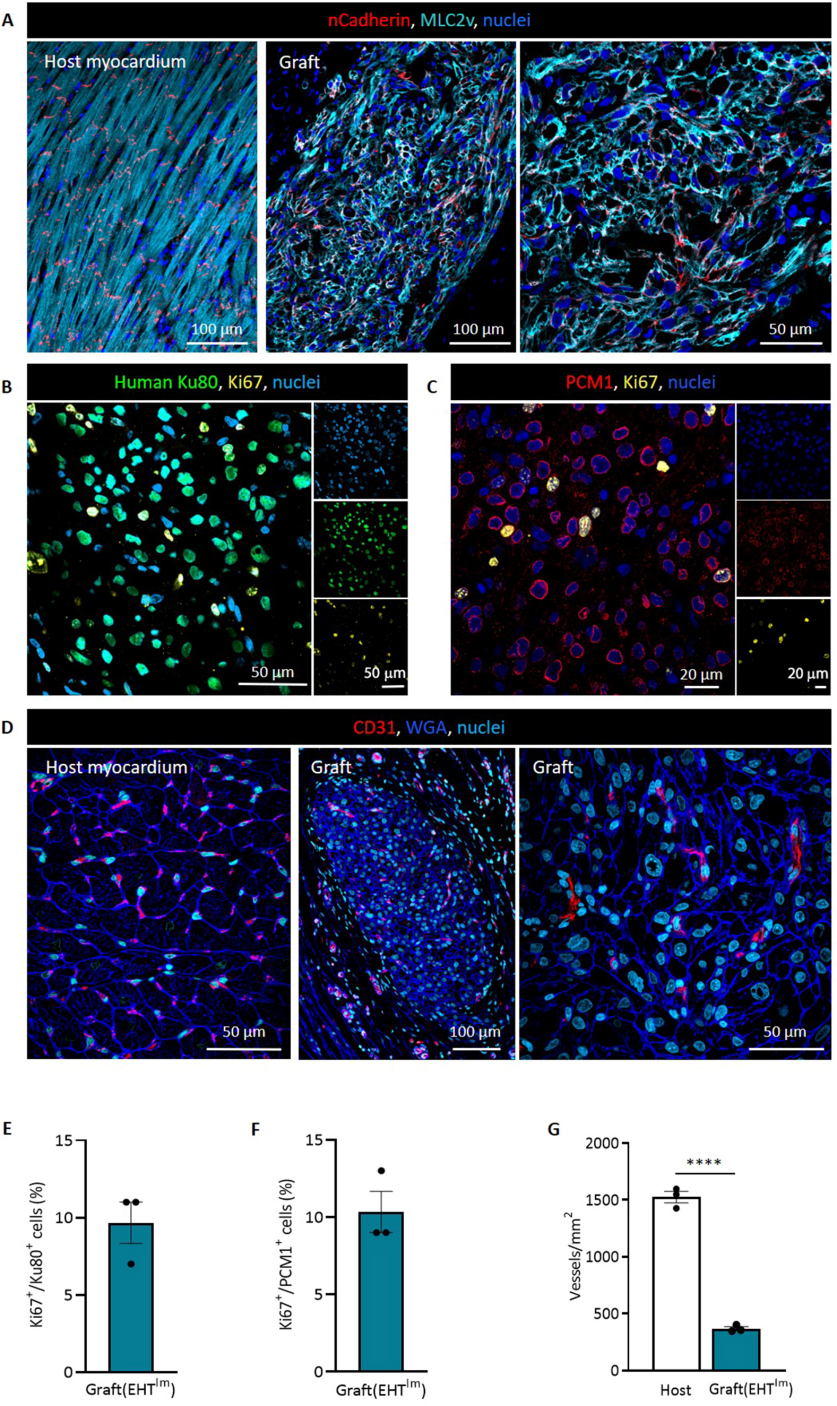
**Analysis of cardiomyocyte proliferation and vascularization.** (A) N-cadherin and MLC2v staining of host and graft tissue. (B,C) Human Ku80/Ki67 (B) and PCM1/Ki67 (C) double immunostaining of engrafted cardiomyocytes. (D) Human graft and host myocardium stained for wheat germ agglutinin (WGA) and CD31 (also known as PECAM1). (E,F) Quantification of Ki67^+^/Ku80^+^ nuclei (E) and Ki67^+^/PCM1^+^ nuclei (F) 4 weeks after transplantation (*n*=3 hearts/three high-magnification images per graft). (G) Quantification of graft vascularization 4 weeks after transplantation (*n*=3 hearts/three high-magnification images per graft or host myocardium). Each data point represents one heart. Mean±s.e.m. values are shown. ****P<0.0001 (two-tailed unpaired *t*-test).

Human grafts consisted of densely packed CMs with advanced sarcomeric structure (sarcomere length, 1.8±0.0 μm; *n*=3; Fig. 2A,D). A subpopulation of the engrafted CMs expressed the immature/fetal atrial isoform of myosin light chain [MLC2a (also known as MYL7), 13±1%; Fig. 2B,E], whereas most cells expressed the ventricular isoform (MLC2v; 87±1%). In contrast to this finding, most cells still expressed the slow skeletal troponin I isoform (ssTnI; 89±1%; Fig. 2C,F). Cardiac troponin I (cTnI; also known as TNNI3) was weakly expressed (10±1%). Additional evidence of immaturity was the circumferential N-cadherin (also known as CDH2) expression (Fig. 3A) and the ongoing cell cycle activity (Fig. 3B,C). Four weeks after transplantation, a subpopulation of engrafted CMs was still in the cell cycle, as indicated by the expression of Ki67 (also known as MKI67) and Ku80 or PCM1 (Ki67^+^/Ku80^+^ nuclei, 10±1%; Ki67^+^/PCM1^+^ nuclei, 10±1%; Fig. 3E,F). Vascularity in the graft was lower than in the remote myocardium (368±17 vessels/mm^2^ versus 1525±50 vessels/mm^2^ in the host myocardium; *n*=3; Fig. 3D,G).

### Functional effects on left-ventricular function

**Fig. 4.**
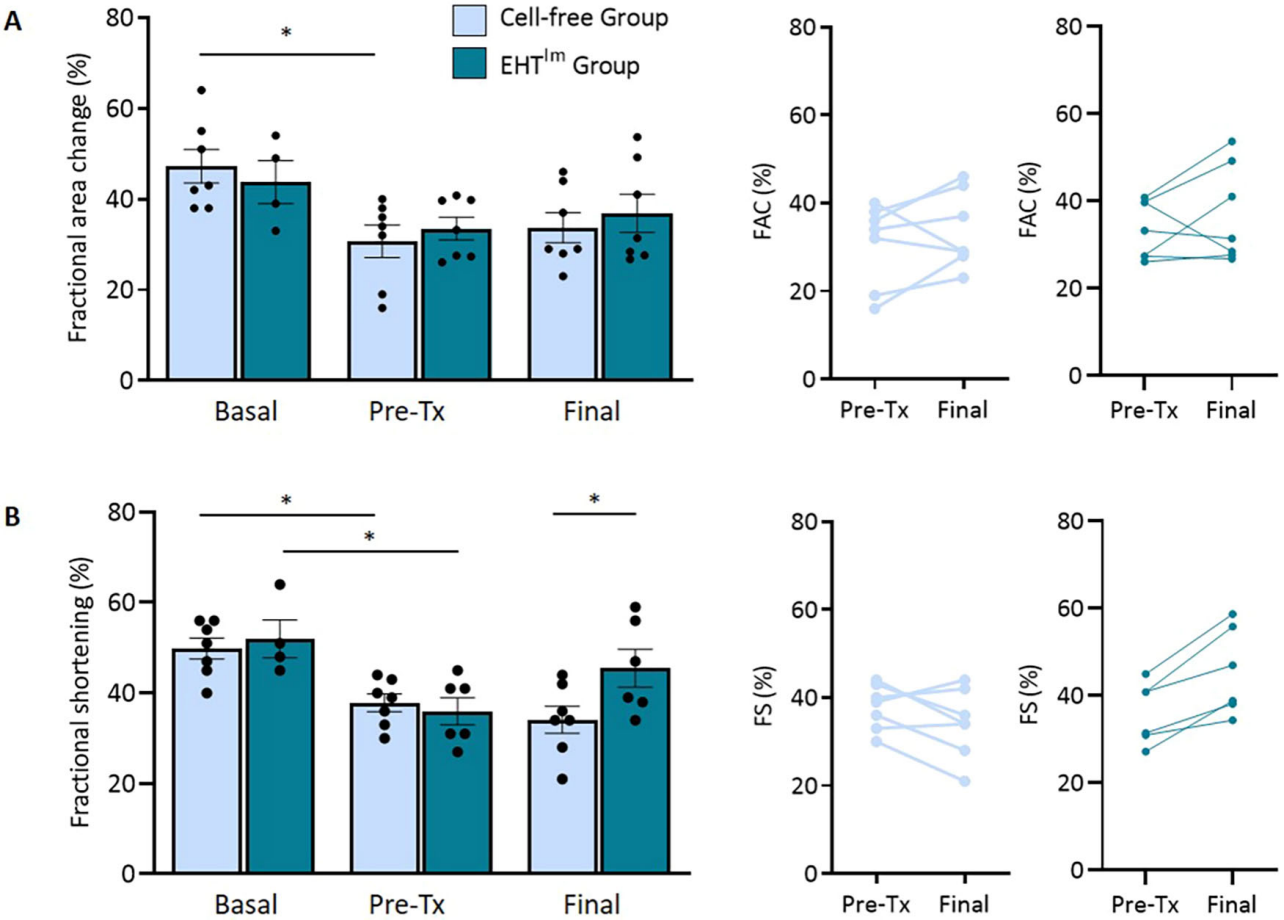
**Echocardiographic evaluation.** (A,B) Fractional area change (FAC; A) and fractional shortening (FS; B) analysis at baseline, 4 weeks after injury and 4 weeks after transplantation (*n*=7 cell-free group, *n*=6 EHT^Im^ patch group) (left), and differences between day 28 (post injury) and 4 weeks after transplantation of the respective groups (middle and right). Mean±s.e.m. are shown, *P<0.05 (two-way ANOVA analyses). Pre-Tx, before transplantation (post injury).

Functional effects of EHT^Im^ patch transplantation were assessed by serial echocardiography (Fig. 4). At baseline, fractional area change (FAC) was 46±3% and fractional shortening (FS) was 51±2%. Myocardial injury resulted in reduction of left-ventricular function 4 weeks after injury (FAC: control group, 31±4% versus EHT^Im^ group, 34±3%; FS: control group, 38±2% versus EHT^Im^ group, 36±3%). FAC showed no improvement in left-ventricular function (FAC: control group, 34±3% versus EHT^Im^ group, 37±4%), whereas FS was higher in the EHT^Im^ group than in animals that received cell-free patches (FS: control group, 34±3% versus EHT^Im^ group, 46±4%).

### Transplantation of large EHT^Im^

**Fig. 5.**
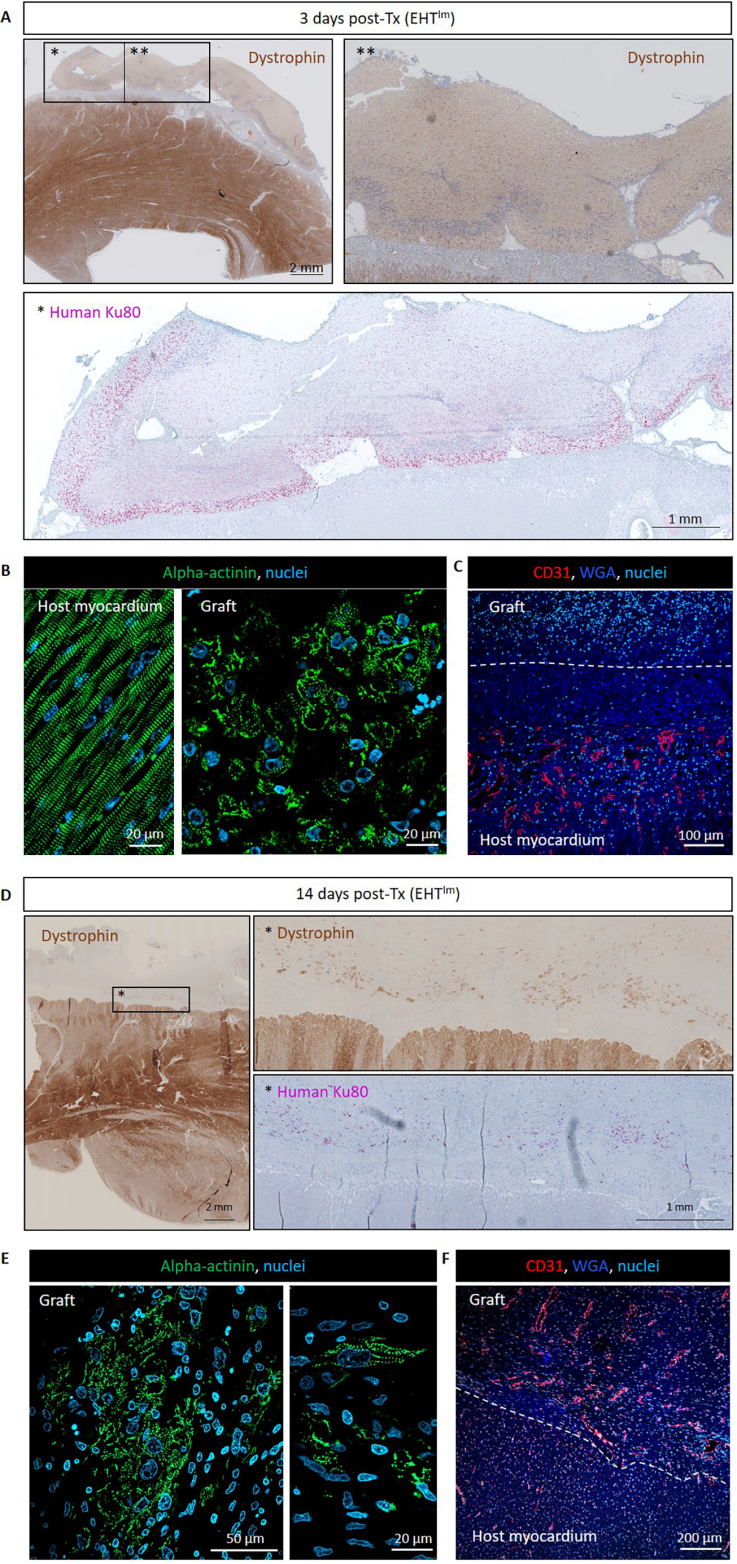
**Histological evaluation after EHT^Im^ transplantation in pigs.** (A) Section of the anterior wall 3 days after EHT^Im^ transplantation stained for dystrophin and human Ku80 (graft shown at higher magnification in * and **). (B,C) Alpha-actinin staining in host and graft myocardium (B), and staining for WGA (wheat germ agglutinin) and CD31 in host myocardium (C). (D) Sections of one heart 2 weeks after transplantation stained for dystrophin and human Ku80 (graft shown at higher magnification in *). (E,F) Alpha-actinin staining in graft myocardium (E), and staining for WGA (wheat germ agglutinin) and CD31 in host and graft myocardium. Post-Tx, after transplantation.

As a further translational step, large EHT^Im^ patches were generated and transplanted on healthy pig hearts (5×7 cm, 4.1-4.8×10^8^ cells; *n*=2 EHT^Im^ patches). Human-scale EHT^Im^ patches were epicardially transplanted in LEA29Y pigs (*n*=2). Animals were sacrificed 3 days and 14 days after transplantation (Fig. S2B). Histological analysis 3 days after transplantation showed a large number of human CMs, mainly localized in the former surface parts of the EHT^Im^ patch (Fig. 5A). Sarcomeric organization was low, and no invasion of vessels was detected in the early days after transplantation (Fig. 5B,C). Two weeks after transplantation, small grafts were visible (Fig. 5D). CM structure and vascularization were more advanced than at the 3 days time point (Fig. 5E,F).

## DISCUSSION

In this study, we investigated the transplantation of EHT^Im^ patches in a guinea pig chronic injury model. Transplantation of hiPSC-derived CMs represents a promising conceptually new therapeutic strategy for heart failure patients (Weinberger and Eschenhagen, 2021). First clinical trials commenced in 2021 (Weller, 2021; Cyranoski, 2018). The clinical trials are based on preclinical studies that repeatedly demonstrated that CM transplantation can improve, or at least stabilize, heart function after injury (Weinberger et al., 2016; Querdel et al., 2021; Shiba et al., 2012; Funakoshi et al., 2016; Laflamme et al., 2007; Liu et al., 2018; Romagnuolo et al., 2019; Chong et al., 2014; Munarin et al., 2020; Zhu et al., 2018; Narita et al., 2017). Yet, most of these studies were performed in subacute injury models, whereas most patients with advanced heart failure suffer from a chronic disease progression (Eschenhagen et al., 2022). Few studies have evaluated CM transplantation in chronic injury models and showed that CM engraftment in the chronically injured heart was less efficient. In a recent study, EHT patch transplantation resulted in a lower degree of remuscularization when targeting the chronically injured heart (von Bibra et al., 2022) than that obtained using transplantation in a subacute situation (Querdel et al., 2021). Likewise, other studies showed that the regeneration of chronically injured heart (Shiba et al., 2014; Fernandes et al., 2010; Riegler et al., 2015), and also spinal cord injuries (Nagoshi et al., 2020; Stevens and Murry, 2018), is more challenging, indicating that this is not a peculiarity of EHT transplantation but a more general problem for regenerative medicine.

Here, we evaluated whether EHT^Im^, i.e. EHTs directly after casting, can be applied to regenerate the chronically injured heart. The study was based on the finding that transplanted matured EHTs showed remarkable dedifferentiation in the first week after transplantation on the injured heart (Querdel et al., 2021). At the time of transplantation, CMs in the matured EHTs morphologically resembled mature CMs (e.g. elongated cell shape, regular sarcomeric structure), but, during the first days after transplantation, most of this structure was lost, and only after 1-2 weeks the surviving CMs recovered, restructured and regained a regular sarcomeric structure. We therefore hypothesized that transplantation of more immature CMs in a three-dimensional tissue will circumvent this step and thereby facilitate clinical translation.

EHT^Im^ engrafted in the chronically injured heart and echocardiographic analysis showed some evidence for improvement in left-ventricular function. Overall, the results were similar to those from our recent study with matured EHTs after a culture period of 3 weeks (von Bibra et al., 2022). Yet, graft size was slightly larger in the current study, which could reflect (1) better engraftment of immature CMs and also (2) the larger cell number of transplanted cells. Even though it is difficult to assess the exact number of vital CMs in the EHT at the time of transplantation, the histological analysis and DNA content measurements indicate that the number of transplanted viable CMs was substantially higher in the EHT^Im^ approach. The engrafted CMs were more immature in the current study, although the differences were small, and there were no differences in sarcomeric structure, cell cycle activity and graft vascularization 4 weeks after transplantation. Although the findings of this study are in line with work on rodent CMs demonstrating that neonatal (immature) CMs survive transplantation whereas adult ones do not (Reinecke et al., 1999), they somewhat contradict our own results that showed a more favorable effect when transplanting more mature EHTs (cultured in the presence of serum) over less mature EHTs that were cultured without serum (Querdel et al., 2021). Retrospectively, the stronger force of EHTs that were cultured in the presence of serum might not have only reflected maturity but also overall cell wellbeing.

A small proof-of-concept study was performed in pigs to evaluate the feasibility of transplanting large EHT^Im^ in a pig model. A previous study revealed technical problems when transplanting smaller matured EHT patches (Querdel et al., 2021). Large EHT^Im^ patches resisted the forces of a pig heart and could be transplanted successfully. Three days after transplantation, the patch was adapted to the epicardial surface but, surprisingly, not vascularized. Two weeks after transplantation, small vascularized grafts were formed.

A limitation of this study is that echocardiographic analysis demonstrated an improvement in FS but not in FAC. This discrepancy probably highlights the technical difficulties in performing echocardiography in this animal model after repeated thoracotomies. Additionally, anterolateral injuries, as induced in our model, can be underestimated by short-axis M-mode imaging (Scherrer-Crosbie et al., 2001; Kanno et al., 2002). Comparison between the current study and previous studies with matured patches is difficult because they most likely differed in input CM number (discrepant CM purity after differentiation and drop in cell number during culture).

In summary, this study shows that transplantation of freshly prepared, EHT^Im^ patches is feasible. EHT^Im^ engrafted in the chronically injured heart. The use of EHT^Im^ comes at the cost of losing contractility of the patches as a central quality criterion, but would reduce practical challenges and costs of cultivating EHTs over extended time prior to transplantation.

## MATERIALS AND METHODS

### Cardiac differentiation and generation of EHT

CMs were differentiated from hiPSCs (UKEi1-A) as previously described (Querdel et al., 2021). UKEi001-A was reprogrammed by a Sendai Virus (CytoTune iPS Sendai Reprogramming Kit, Thermo Fisher Scientific). EHT patch casting was performed by mixing CMs and a fibrinogen/thrombin mix (Weinberger et al., 2016). EHT patches (1.5×2.5 cm) were generated with 15×10^6^ hiPSC-derived CMs per 1.5 ml casting medium for the guinea pig study. EHT^Im^ patches were subsequently transplanted within 2-5 h after casting. Control animals received cell-free patches. For the pig study, large, human-scale EHT patches (5.0×7.0 cm) with 4.1-4.8×10^8^ cells were cast. Cells were resuspended in 25 ml casting medium. The human-scale patches were cast at the University Medical Center Hamburg-Eppendorf, transported to the Technical University Munich and transplanted within 12 h after casting.

### Cell content analysis in EHT and EHT^Im^ patches

After casting, EHT patches were either directly harvested (EHT^Im^) or cultured for 3 weeks prior to harvest (EHT). To assess cell numbers, EHT^Im^ and EHTs were processed for histology, and serial sections were acquired. One section from the upper surface, one section from the middle section and one section from the lower surface were analyzed. Assuming that each CM was mononucleated, that CM percentage does not change substantially over the culture period (Querdel et al., 2021) and that the EHTs contained only CMs (neglecting the ∼5% troponin t^−^ cells after differentiation), the number of nuclei was regarded as CM number. Additionally, total DNA content was used to estimate cell content. For this, EHT^Im^ and EHTs were snap frozen, and DNA was isolated with Trizol (Invitrogen) and quantified by a spectrophotometer (Nanodrop One, Thermo Fisher Scientific).

### Animal care and experimental protocol approval

The investigation was performed in accordance with the guide for the care and use of laboratory animals published by the National Institutes of Health (Publication No. 85-23, revised 1985) and was approved by the local authorities (Behörde für Gesundheit und Verbraucherschutz, Freie und Hansestadt Hamburg 109/16 and Regierung von Oberbayern 02-18-134).

### Guinea pig chronic injury model and EHT transplantation

Female Dunkin Hartley guinea pigs were used (400-460 g, 6-8 weeks of age, Envigo) to induce chronic myocardial injury (von Bibra et al., 2022). Cryo-injury of the left ventricular wall was performed with a liquid nitrogen-cooled probe. Four weeks after injury, a rethoracotomy was performed, and the cryo-lesion was covered with an EHT^Im^ patch (15×10^6^ hiPSC-derived CMs) or a cell-free patch. The study was performed overlapping in time with a previously published study in which matured EHTs were transplanted (von Bibra et al., 2022). Animals that received cell-free patches served as control group for both studies. All animal procedures were performed by the same animal surgeons (C.v.B. and B.G.). Guinea pigs were immunosuppressed from 2 days prior to transplantation with cyclosporine (7.5 mg/kg for the first 5 postoperative days and 5 mg/kg per day over the follow-up period of 4 weeks; mean plasma concentration, 562±87 µg/l; *n*=7) and methylprednisolone (2 mg/kg). Cyclosporine plasma concentration was measured from whole-blood samples with the laboratory's routine liquid chromatography–tandem mass spectrometry method.

### Echocardiography

Cardiac function was assessed by transthoracic echocardiography (Castro et al., 2019) with a Vevo 3100 System (Fujifilm VisualSonics). Echocardiographic recordings were taken at baseline, 4 weeks after cryo-injury and 4 weeks after transplantation. Because of technical issues with the system, three animals did not receive baseline echocardiography. One animal was excluded from the functional analysis because of inadequate image quality.

### Heart preparation and histology

Four weeks after transplantation, hearts were processed for histology (Querdel et al., 2021). Hearts were fixed in formalin for 2 days, sliced into four cross-sections and paraffin-embedded for histology. Images of dystrophin- and human Ku80-stained slides were acquired with a Hamamatsu Nanozoomer whole-slide scanner and analyzed with NDP software (NDP.view 2.3.1). Dystrophin-stained sections were used to determine infarct size and graft area (Weinberger et al., 2016; Querdel et al., 2021). Grafts were recognized by human Ku80 staining. To assess CM density, human Ku80^+^ nuclei were automatically counted (Q-Path 0.2.3) in 200 µm×200 µm fields. Human Ku80^+^ nuclei quantification was performed in the section that contained the largest graft. The primary antibodies used are listed in Table S1. Immunofluorescence images were acquired with an LSM 800 confocal microscope (Zeiss).

### EHT^Im^ transplantation onto healthy pig heart

Transgenic pigs overexpressing a human CTLA4-Ig derivate (LEA29Y) were used for the transplantation study (Bähr et al., 2016). LEA29Y pigs were anesthetized by intramuscular injection of ketamine, azaperone and atropine sulfate, and mechanically ventilated. Anesthesia was maintained by a constant intravenous application of propofol and fentanyl. EHT^Im^ patches were transplanted through a left-sided lateral thoracotomy (Querdel et al., 2021). One large, human-scale EHT^Im^ patch per heart was sutured onto the healthy epicardium. LEA29Y pigs were additionally pharmacologically immunosuppressed with methylprednisolone (250 mg on the day of surgery and 125 mg/day starting at day 1 after surgery), tacrolimus (0.2 mg/kg/day) and mycophenolate mofetil (40 mg/kg/day). Animals were sacrificed under anesthesia by intravenous injection of saturated potassium chloride (3 days and 14 days after transplantation).

### Statistical analysis

Statistical analyses were performed with GraphPad Prism 8.3.0 or 9.3.1. Comparison among two groups was made by two-tailed unpaired Student's *t*-test. One-way ANOVA followed by Tukey's test for multiple comparisons were used for more than two groups. When two factors affected the result (e.g. time point and group), two-way ANOVA analyses and Tukey's test for multiple comparisons were performed. Error bars indicate s.e.m. *P<0.05 was considered significant.

This article is part of a collection ‘Moving Heart Failure to Heart Success: Mechanisms, Regeneration & Therapy’, which was launched in a dedicated Special Issue guest edited by Jeroen Bakkers, Milena Bellin and Ravi Karra. See related articles in this collection at https://journals.biologists.com/collection/8169/Moving-Heart-Failure-to-Heart-Success.

## Supplementary Material

10.1242/dmm.049834_sup1 Supplementary information
