# Supplementary material for: Immature human engineered heart tissues engraft in a guinea pig chronic injury model
Source: Dis Model Mech. 2023 Jun 5;16(5):dmm049834. doi: 10.1242/dmm.049834 (PMC10259837; doi:10.1242/dmm.049834)
Supplement: Supplementary information [file dmm-16-049834-s1.pdf]

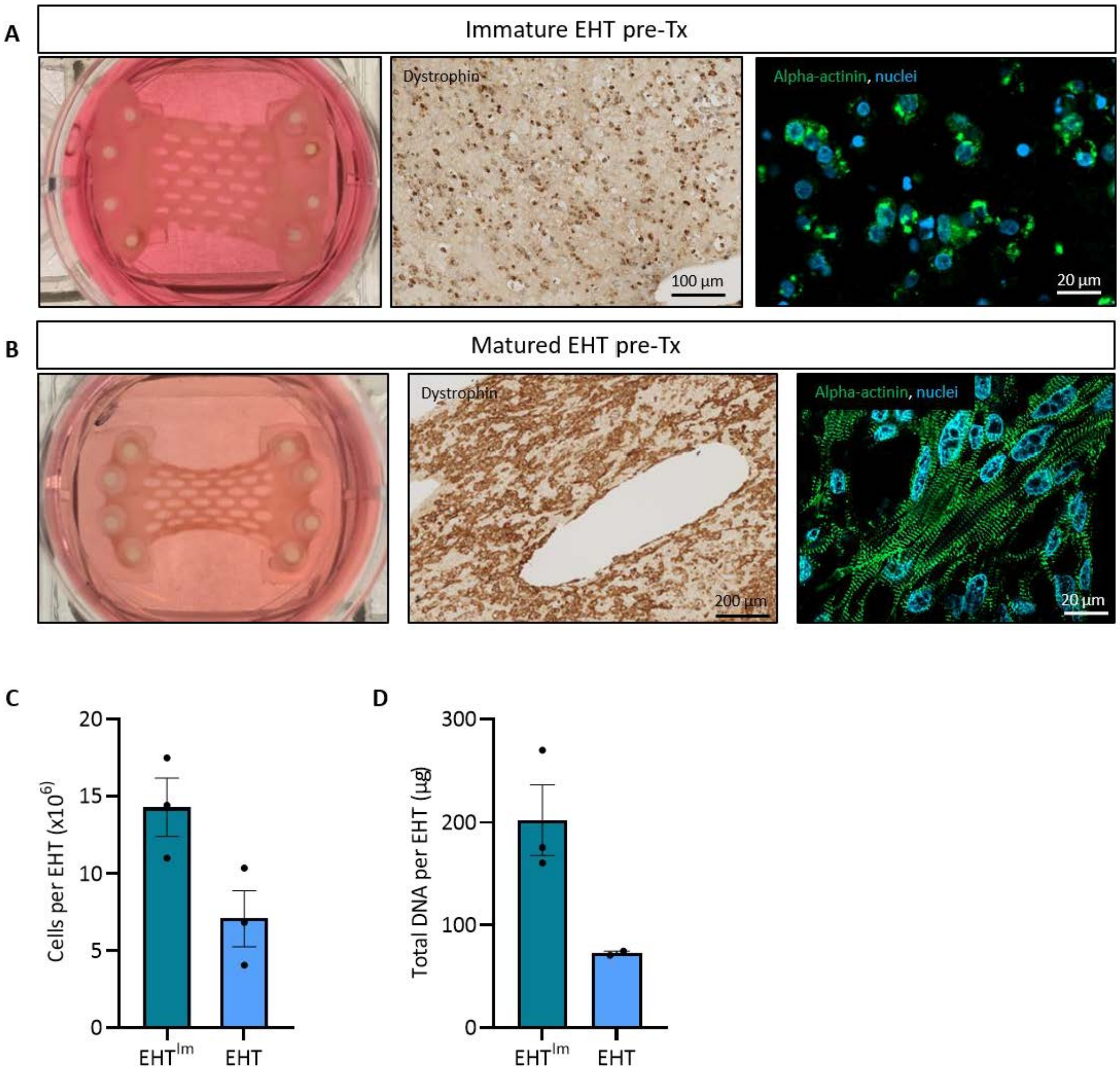

Fig. S1. Development of engineered heart tissue. A) Photograph of an immature EHT at the time of casting (before transplantation) and dystrophin and alpha-actinin stained sections. **B)** Photograph of an EHT after 3 weeks in culture (before transplantation) and dystrophin and alpha-actinin stained sections. **C)** Quantification of cell number and **D)** DNA content per EHT^{Im} and matured EHT. Each data point represents one EHT. Mean ± SEM are shown. EHT indicates engineered heart tissue; Pre-Tx, before transplantation**.**

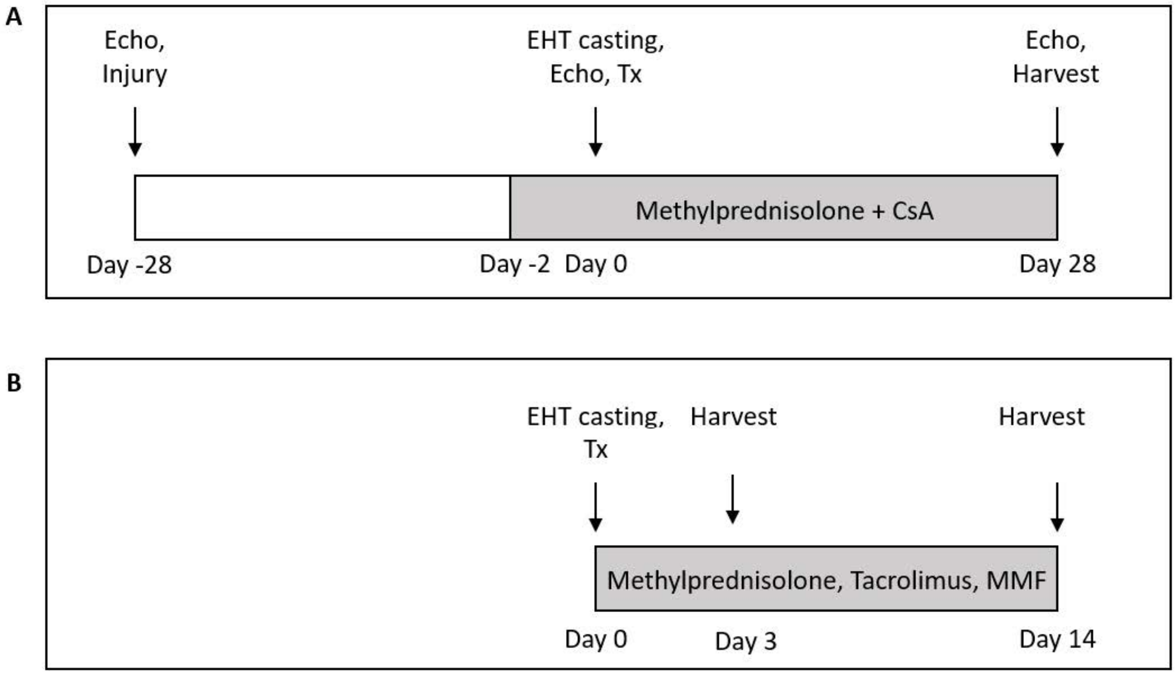

Fig. S2. Study protocols. A) Experimental protocol for the small animal study. **B)** Study protocol for the proof-of-concept study in pigs. Echo indicates Echocardiography; EHT, engineered heart tissue; Tx, transplantation; CsA, cyclosporine and MMF, mycophenolate mofetil.

Disease Models & Mechanisms • Supplementary information

Table S1. Primary antibodies for histology

Antigen	Vendor	Antigen retrieval	Titer
**MLC2a**	BD Pharmingen, clone S58-205, Cat. 565496	Citrate buffer	1:500
**MLC2v**	Proteintech, pAb, Cat.10906-1-AP	Citrate buffer	1:250
**α-actinin**	Sigma-Aldrich, clone EA-53, Cat. A7811	Proteinase K	1:600
**Ku80**	Cell Signaling Technology, clone C48E7, Cat. CST-2180	Citrate buffer	1:800
**N-cadherin**	Sigma-Aldrich, clone CH19, Cat. C1821	Citrate buffer	1:400
**ssTnI**	Novus Biologicals, clone OT18H8	Citrate buffer	1:100
**cTnI**	Abcam, Cat. Ab47003	Citrate buffer	1:100
**PECAM1 (CD31)**	Novusbio, Cat. NB 100-2284	Citrate buffer	1:100
**Ki67**	Invitrogen, clone Sol-A15, Cat. 2196793	Citrate buffer	1:400
**PCM1**	Sigma, 023370	Citrate buffer	1:100

Disease Models & Mechanisms • Supplementary information
